# Characteristics of phantom upper limb mobility encourage phantom-mobility-based prosthesis control

**DOI:** 10.1038/s41598-018-33643-0

**Published:** 2018-10-18

**Authors:** Amélie Touillet, Laetitia Peultier-Celli, Caroline Nicol, Nathanaël Jarrassé, Isabelle Loiret, Noël Martinet, Jean Paysant, Jozina B De Graaf

**Affiliations:** 1 IRR Louis Pierquin – UGECAM Nord-est, 75 Boulevard Lobau, CS 34209, 54042 Nancy Cedex, France; 2 Development, Adaptation and Handicap, University of Lorraine, 30 Rue du Jardin Botanique CS 30156, 54603 Nancy, France; 3 Aix Marseille Univ, CNRS, ISM, Marseille, France; 4 Sorbonne Université, CNRS, INSERM, Institut des Systèmes Intelligents et de Robotique, ISIR, 75005 Paris, France

## Abstract

There is an increasing need to extend the control possibilities of upper limb amputees over their prosthetics, especially given the development of devices with numerous active joints. One way of feeding pattern recognition myoelectric control is to rely on the myoelectric activities of the residual limb associated with phantom limb movements (PLM). This study aimed to describe the types, characteristics, potential influencing factors and trainability of upper limb PLM. Seventy-six below- and above-elbow amputees with major amputation underwent a semi-directed interview about their phantom limb. Amputation level, elapsed time since amputation, chronic pain and use of prostheses of upper limb PLM were extracted from the interviews. Thirteen different PLM were found involving the hand, wrist and elbow. Seventy-six percent of the patients were able to produce at least one type of PLM; most of them could execute several. Amputation level, elapsed time since amputation, chronic pain and use of myoelectric prostheses were not found to influence PLM. Five above-elbow amputees participated in a PLM training program and consequently increased both endurance and speed of their PLM. These results clearly encourage further research on PLM-associated muscle activation patterns for future PLM-based modes of prostheses control.

## Introduction

Given the development of new biomimetic devices with numerous active joints (e.g. recent polydigital hands), there is an increased need to extend the control possibilities of upper limb amputees over their prosthetics^1^. Invented in the fifties^2^ and still implemented in current prostheses (including the new polydigital models), myoelectric control associates the surface myoelectrical activities (EMG) from the residual limb to one or several prosthetic movements. An on/off strategy is applied by thresholding the input signals (amplitude and temporal EMG variations) that the patient needs to produce with the concerned muscle(s). Often, each active prosthetic joint that composes the substituting limb is sequentially controlled by the same control inputs. So, despite the potential possibilities offered by new biomimetic prostheses such as whole robotic arms [DEKA Luke ARM]^3^ or polydigital hands, their control is still complex (far from intuitive) and offers few functional degrees of freedom^1^.

To overcome these limitations, pattern-recognition approaches were developed in the late 60 s/70s^4–6^, with the aim of decoding more precisely the myoelectric signals and thus to recognize more classes of contractions from the EMG signals and to control more classes of motions. This requires the use of multiple measurement sites, more precise extraction of signal information (not only the amplitude), and multidimensional classification architecture. While well established and extensively studied, such approaches have only recently been applied commercially to prosthetics control (see the COAPT system, http://www.coaptengineering.com/), notably due to issues with limitations in clinical robustness^7^ (pattern variability, noise, sensitivity to numerous external factors like muscle fatigue, electrode shift or skin variations, etc.).

One way of feeding pattern-recognition myoelectric control is to rely on the EMG activities of the residual limb associated with phantom limb movement (PLM) execution. Voluntary PLM have recently been shown to be a form of “real” motor execution^8–10^, with underlying neurophysiological mechanisms different from those of motor imagery^11,12^. The associated muscle activity varies with the type of executed PLM^9,11,13^ even for different finger movements in above-elbow amputees^13^. This pattern-recognition approach has been extensively studied for below-elbow amputees whose residual limb usually contains muscles that mobilized the fingers before the amputation, and, therefore, provide an adapted measurement site together with relatively strong EMG signals. While numerous adaptations of these approaches were made to above-elbow amputees in the 70 s/90s^14–16^, it is the development of targeted-muscle-reinnervation approaches^17^ which has made this technique more viable and transferrable to patients^18^. Even so, several studies have recently revived this approach in amputees without targeted reinnervation, using only natural phantom-limb-mobility-related residual EMG signals^13,19–21^.

In the case of transhumeral amputation, PLM related EMG activity must be measured over muscle groups of the residual limb which -before amputation- were not mechanically acting on the joints of the missing limb. These signals, whether due to neuro-muscular reorganization or to remaining global supporting contraction schemes (proximal residual muscles acting in synergy with movements of the -now missing- distal limb), still seem to contain information regarding PLM. But these signals remain inevitably more complex and challenging to decode in daily life, especially without reinforcing these PLM related EMG signals through muscle reinnervation surgery^17^. Nevertheless, despite the development of more natural prosthetic control approaches based on PLM use/decoding^13,19,22,23^, little is known from an epidemiological point of view.

As early as in 1948, Henderson and Smyth^24^ reported the presence of voluntary PLM in most patients. Since then, PLM has received limited attention in the literature for three reasons. First, the phantom limb is widely considered as associated to phantom pain and studied as such^25^. The few studies mentioning PLM mostly focus on phantom limb pain and sensations^26–30^ and use a rather inhomogeneous and small population. Second, patients are not encouraged by rehabilitation staff to explore their PLM for fear of disturbing prosthesis control^26^. Third, many patients and health professionals still believe that PLM are the “fruit of a highly active imagination”, reflecting “the non-acceptance of the limb loss”^26^, and PLM are often considered as imaginary movements despite recent neurophysiological evidence to the contrary^9,10^. Quantitative information is thus lacking on the percentage of patients with voluntary control of PLM and on the PLM evolution over time. No study has provided an exhaustive description of the panoply of PLM which patients can execute.

The underlying assumption of the present study was that the more controllable the phantom limb (i.e., with the ability to move different joints), the higher the probability that associated muscle activation patterns are strong and contain characteristic information on the PLM which can then be robustly decoded. Thus, the first aim was to quantify the exact occurrence of PLM among upper limb amputees without targeted muscle-reinnervation^17^. The second aim was to precisely describe PLM types, characteristics and potential influencing factors via semi-directed interviews of upper limb amputees.

The few studies^12,31^ that have explored kinematic aspects of PLM have shown that PLM are generally slow, and perceived as effortful and difficult, which might be contradictory to their eventual use for prosthesis control. However, most patients spontaneously declared that training phantom movements would make their execution easier^31^. Indeed, PLM training has already been studied, but only for below-elbow amputees^23^ or for release of phantom pain^32,33^. Therefore, the third aim of this study was to compare kinematic aspects of PLM before and after a daily training of 1 to 2 months in 5 above-elbow amputees without phantom and residual limb pain.

## Results

### Global phantom limb sensations and mobility

Seventy out of the 76 patients (92%) described various painless phantom sensations such as general awareness and non-painful somatic sensations (warmth, cold, pressure, tingling…), all of them at least involving the fingers and often the whole hand. At the time of the interview, 76% of all patients (31 out of 37 above-elbow amputees and 27 out of 39 below-elbow amputees) described the ability to produce voluntary PLM. Only 16% of them had never produced any PLM, whereas 8% described an initial but temporary capacity to produce voluntary PLM.

Forty-four patients (59% of all patients) described pain: for 14 patients, it concerned isolated phantom limb pain, 12 patients experienced isolated residual limb pain, and 18 reported both phantom and residual limb pains. Ten patients reported permanent pain (4 isolated phantom limb pain, 2 isolated residual limb pain, and 4 pain in both). These same 10 patients could produce several types of voluntary PLM. Eight patients reported either pain appearance or increase during voluntary PLM. Only 1 patient described an increased difficulty to produce PLM when phantom limb pain was present. Two patients reported muscle pain in the residual limb (with no phantom limb pain) while repeating voluntary PLM. On the other hand, 4 patients reported using voluntary PLM to relieve phantom limb pain. Four others mentioned that an initially painful and frozen closed phantom hand was relieved after training the phantom hand to open. So, different interactions between pain and PLM were found, but the existence of pain does not exclude voluntary PLM.

Twenty-six of the patients described that repetition of voluntary PLM generated fatigue, eventually leading to a progressive or sudden blocking of the PLM (the phantom limb was perceived as paralyzed and when the patient still tried to perform the given PLM, it was felt as “clamped in a vice”) without associated pain sensations. The perceived effort was often restricted to only some types of PLM rather than a general phenomenon, but the types of voluntary PLM that generated fatigue largely varied amongst patients.

### Characteristics and types of phantom limb movements

For all patients who could mimic the voluntary PLM with their intact limb, the movements were smaller and slower than natural intact limb movements. Yet, the different PLM were clearly distinct from each other. It is interesting to note that the use of a standardized interview encouraged the patients to explore their mobility capacity. Some patients discovered during the interview additional PLM since they “don’t execute PLM in daily life”.

**Figure 1:**
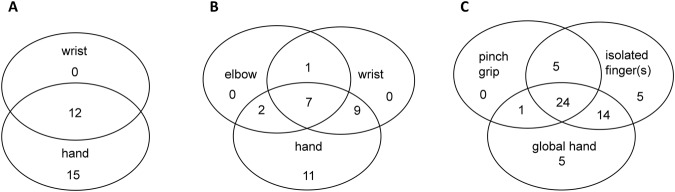
Number of patients with PLM at the indicated phantom limb level(s). (**A**) Results for the PLM of below-elbow amputees. (**B**) Results concerning the above-elbow amputees. Note that no patient had PLM exclusively at the wrist or elbow. (**C**) Results for the phantom hand for all below and above-elbow amputees except for 3 patients with only phantom finger abduction and adduction movements.

**Figure 2:**
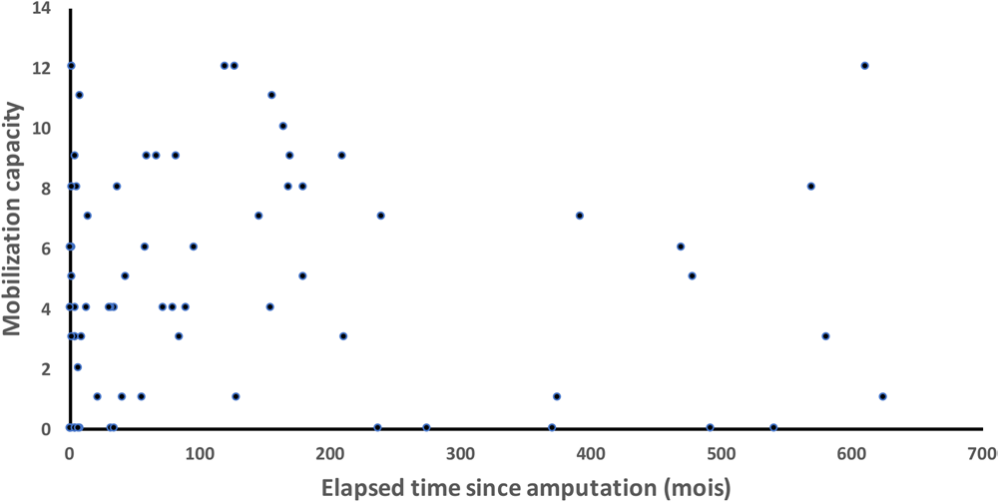
Mobilization capacity (i.e. number of distinct types of phantom movements) for the phantom hand and wrist as a function of time elapsed since amputation (in months) for all 76 patients included in the study. No relation between the two variables was found (r = 0.0118, p > 0.8).

Thirteen predetermined distinct types of voluntary PLM were identified (Table 1). Nine distinct hand PLM were reported, including global hand opening/closing, flexion/extension of individual fingers, pinch grip closing/opening (opposing the thumb to the index or sometimes to another finger), flexion/extension of 2 to 3 undissociated fingers, and abduction/adduction of the fingers. Eight patients could perform all of them. All 3 degrees of freedom of the wrist PLM (flexion/extension, ulnar/radial inclination and prono-supination) were reported; 11 patients were able to do all of them. Five below-elbow patients could perform the 12 hand and wrist PLM (Figs 1A and 2) while only 10 of the 37 above-elbow amputees could perform phantom elbow flexion/extension movements (Fig. 1B). For each patient, the total number of different voluntary PLM over all segments will be called “mobilization capacity”.

**Table 1:** Number of participants who could produce the given type of phantom limb movements. Many patients could produce several types.

Types of phantom limb movements	Number of patients
Isolated flexion/extension from at least one to five fingers	48
Pinch grip opening/closing	29
Flexion/extension of 2 or 3 undissociated fingers	17
Finger abduction/adduction	19
Global hand opening/closing	44
Forearm prono-supination	21
Wrist flexion/extension	22
Ulnar/radial inclination	18
Elbow flexion/extension	10

More patients were able to mobilize the phantom hand than wrist and elbow (see Table 1 and Fig. 1). If PLM were possible at proximal parts of the phantom limb, the hand was generally also mobile. Indeed, only 1 above-elbow amputee had voluntary PLM at the wrist and elbow, but none at the hand (Fig. 1B). None of the below-elbow amputees could only move the wrist without being able to move at least 1 finger (Fig. 1A). Hand and wrist mobilization capacities were not influenced by the amputation level: a group-comparison between above- (n = 37) and below-elbow (n = 39) amputees revealed no significant inter-group difference (Mann-Whitney test, U = 696, p > 0.79). This was confirmed by Fisher’s exact test (p > 0.1; power 23.5%).

### Focus on hand phantom movements

Most patients were able to make several types of hand PLM (see Fig. 1C). For patients who could make a voluntary pinch grip (involving thumb and index) and a global hand opening/closing, both involving several fingers at the same time, we evaluated whether isolated movements composing the given combined movement were always possible. This was not the case, although all fingers were clearly perceived. Indeed, 4 patients who could make a pinch grip could individually move only one of the two fingers. One patient even made a pinch grip without being able to make any isolated index or thumb movement. Similarly, isolated finger movements were not always possible although the patient could open/close the global hand. Of the 44 patients who could perform global hand opening/closing movements, only half of them made a pinch grip. The inverse was also found: 16 patients could individually move their index and thumb but were unable to make a pinch grip involving both fingers together.

### Time elapsed since amputation

At less than 6 months post-amputation, 13 of the 21 interviewed patients could produce PLM. They estimated the first PLM occurrence as varying between soon after waking-up (1 patient), during the first weeks (5 patients), during the first month (4 patients) and after 3 to 4 months (3 patients). Another of these patients reported that he had been able to execute PLM during several weeks before subsequently losing this capacity. The patients interviewed in a later phase after amputation equally dated the first PLM to within a few weeks or months after amputation. Figure 2 shows the mobilization capacity of the phantom hand and wrist as a function of the time elapsed since amputation for all patients (n = 76). Statistical analyses revealed no relation between these two variables (r = 0.0118, P > 0.8), suggesting that the time elapsed after amputation does not influence the mobilization capacity. All patients clearly stated that they didn’t execute PLM in daily life, except the 4 patients who produced PLM to alleviate pain.

### Prosthesis use

Eighty-three percent of the interviewed patients used a prosthesis, of whom 62% used daily for more than 6 hours a day. Fifteen percent used a mechanical prosthesis, 12% an esthetical and 47% a myoelectric one, and for 29% the prosthesis was in the fitting process. Nine percent of the patients used several types of prostheses. Only 3 patients mentioned a discomfort related to their phantom limb caused by dissociation in space between their phantom limb and the prosthesis; 2 of them didn’t use their prosthesis anymore. The third patient, interestingly, trained his phantom limb to actively place it into the prosthesis.

The patients using a myoelectric prosthesis reported that the muscular control of their prosthesis was clearly different from the control of voluntary PLM and that they never got mixed-up. We did not find a significant difference in phantom mobilization capacity between users of myoelectric prostheses (n = 36) and patients without myoelectric prosthesis (n = 40) (Mann-Whitney test, U = 543, p > 0.3).

### Training effects

**Figure 3:**
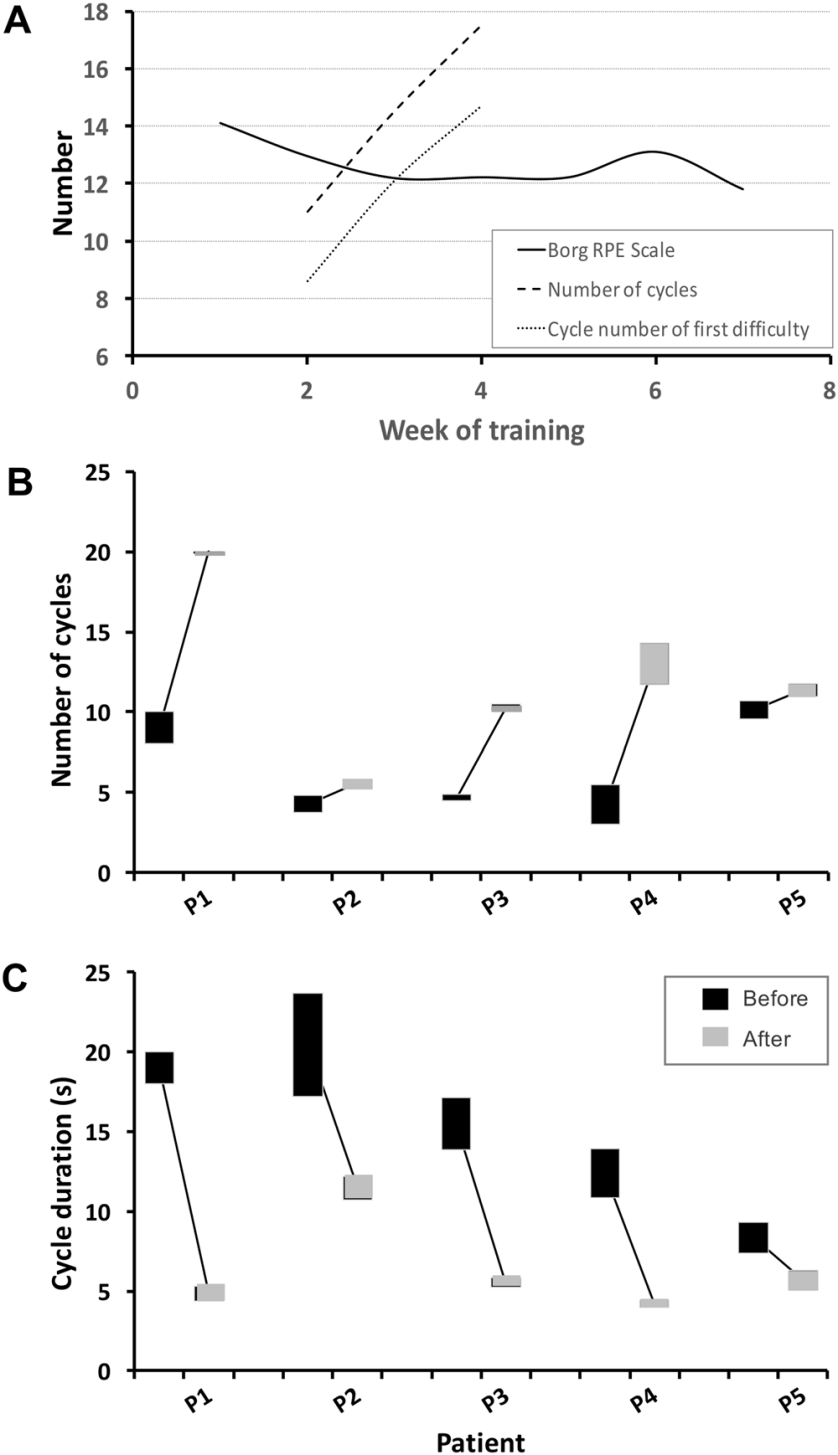
Results concerning the daily PLM-training. (**A**) Evolution of perceived difficulty in PML execution during the training period, successively averaged over 1 week of training and over all types of PLM. Signification of curves is given in the inset. RPE scaling was done by P3, and the number of executed cycles as well as the cycle number of first difficulty by P5. (**B**) Number of cycles that could be executed subsequently before movements blocked due to fatigue, averaged over all types of PLM for each patient (the vertical size of the bars represents the standard error). (**C**) Cycle duration averaged over all types of PLM for each patient (the vertical size of the bars represents the standard error).

For all five trained patients, the weekly phone interview revealed a decrease in the reported difficulty of PLM execution during their daily training period of 1 to 2 months. Two of the patients (P3 and P4) provided a quantified feedback about their sensations for each type of PLM during the training period (Fig. 3A). Patient P3 scored his estimated difficulty in PLM execution (solid curve) during 7 weeks of training, on a Borg Rating of Perceived Exertion (RPE) Scale (from 6 to 20)^34^. Patient P4, who found the Borg rating somewhat difficult, counted during his 4 training weeks the number of cycles he could execute before the PLM blocked (dashed curve) and the first cycle when he felt the execution was becoming difficult (dotted curve). For both patients, the sensation of difficulty was delayed.

After training, none of the five patients reported any pain appearance in either phantom or residual limb. The number of different types of PLM they could execute did not significantly change (N = 5, Z = 1.826, p > 0.05), but the average number of PLM repetitions increased (N = 5, Z = 2.023, p < 0.05) (Fig. 3B) and the cycle duration became shorter (N = 5, Z = 2.023, p < 0.05) (Fig. 3C), meaning an increased speed of execution. All 5 patients reported that PLM had become easier. For some patients, increasing residual muscle contraction (visible by the experimenter from tremor or expanding contractions of the residual muscles) preceded the PLM blocking and usually came at the same time as the execution difficulty, as confirmed by the patient afterwards. After training, this increasing residual limb contraction and the reported difficulty occurred later during the movement sequences.

To evaluate whether the PLM normalized after training, two patients (P3 and P5) were asked to perform at maximal speed with their intact limb 10 cycles of one PLM. P5 made 10 wrist flexion/extensions at a mean cycle duration of 1 s. His corresponding PLM cycle durations were 5.7 s before and 3.8 s after training. P3 executed 10 pinch opening/closings at a 0.7 s mean cycle duration. The corresponding PLM values were 14.2 s before and 4.8 s after training.

Finally, to evaluate the possible influence on performance of the elapsed time between the pre- and the post-training, we compared the pre-training performance of two patients (P3 and P5) to their very first test values (recorded 12 and 30 months earlier, respectively). For patient P3, both the number of cycles and the cycle duration did not differ significantly between the two sessions, as tested with a Wilcoxon test for paired samples, taking the individual types of PLM as cases and the two sessions as repeated measures (N = 5, Z = 1.826, p > 0.06 for number of cycles; N = 5, T = 1, Z = 1.753, p > 0.07 for cycle duration). For patient P5, only the cycle duration was available (he had initially been asked to stop PLM execution after 5 cycles for technical reasons) which equally did not differ between the two sessions (N = 6, Z = 0.943, p > 0.3). Such comparison was not possible for the other 3 patients whose first testing session was their pre-training session. Although limited, these results suggest that PLM training, rather than time, gave rise to the delayed difficulty-onset and the improved PLM performance.

## Discussion

To our knowledge, this is the first quantitative study with a special focus on the occurrence and characteristics of voluntary PLM in a large population of upper limb amputees. Before discussing the results, a methodological remark is perhaps required. The lack of visible movement could have been expected to make the identification of the PLM difficult. However, most of the patients who were asked to verbally describe the movements were surprised at how easily they could do so. Moreover, the patients naturally imitated their PLM with their intact limb, a technique already used in other recent studies^11,12,31^. The combination of both imitation and verbal description appeared adequate for exploring PLM.

The present study reveals that 76% of the patients produced at least one type of PLM at the time of the interview (83% of patients with a phantom limb) and 84% had been able to do so at some point after amputation. One reason for the low percentage of occurrence previously reported in the literature might be that these studies^26–30^ focused on painful and non-painful phantom limb sensations rather than on its voluntary mobility. Moreover, if patients do not experience phantom pain, they do not spontaneously bring up phantom sensations, and even less so phantom mobility^26^.

In contrast with some reports^11,24,29^, but in line with our previous study^31^, the present results show no influence of elapsed time since amputation on mobilization capacity. Only 6 patients reported that movement capacity had ceased after a while, either because of phantom limb disappearance or “paralyzed” phantom limb. Reilly and colleagues^11^ also reported secondary paralysis of the phantom limb. Twenty-two of our 29 patients who were interviewed more than 10 years after amputation, showed PLM. This persistence of PLM capacity is coherent with that reported for one patient at more than 40 years after amputation^12^. Only 4 of our patients intentionally preserved their PLM by regular practice whereas the others reported “not seeing any use of it”. To date, it is not clear why some patients lose their PLM-ability whereas others keep it for a lifetime even without training.

The second aim of this study was to explore characteristics, types and potential influencing factors of the different PLM. As already reported^9,11,12,31,35^, most of the patients performed slower PLM and of smaller amplitudes compared to the natural movements of their intact limb. In agreement with previous reports^11,30,31,36^, some patients showed that for repetitive PLM, the speed and amplitude were progressively limited by fatigue, eventually leading to a blocking of the PLM. Several reports found slower movements when phantom pain was present^11,36–38^, but the present study demonstrates in a large population that the occurrence of phantom pain does not prevent PLM. So, pain seems to influence the PLM quality rather than the variety of PLM a patient can produce. This is interesting since recent studies^33,38^ have shown that PLM can alleviate pain, which is an important point for the potential use of phantom movements in myoelectric prosthesis control.

Although the number and types of voluntary PLM was highly variable amongst patients, the most distal parts of the phantom limb were generally found to be mobile. Wrist movements occurred less frequently, and mostly in patients who could also make phantom hand movements. As previously described^24^, a few patients could produce voluntary elbow movements. Only patients who could mobilize the phantom wrist and/or hand could also produce elbow movements. It has already been shown that phantom limb sensations mostly concern fingertips and parts around joints^24^. The present results show the same predominance of distal parts for the motor control of the phantom limb, with preservation of thumb movements, pinch grip and global hand movements even long after amputation, which is positive for considering PLM for controlling prostheses. Yet, the fact that combined finger PLM (pinch grip, global hand closing/opening) is not just the sum of motor control of individual fingers must be considered for polydigital myoelectric hand control allowing several types of grips.

The present study did not reveal any relation between mobilization capacity and the level of amputation. As mentioned in the introduction, one could have expected a higher occurrence of voluntary PLM in below-elbow amputees than in above-elbow amputees since after a below-elbow amputation, the forearm muscles, which are the most important effectors of many finger and wrist movements, are still (partly) present. The absence of such difference emphasizes that the presence of muscles previously involved in a given movement is not necessary for voluntary PLM, which is a positive point for PLM-based polydigital hand prostheses in above-elbow amputees.

In our population, the reason for consultation was often related to the fitting or maintenance of a (myoelectric) prosthesis, leading to an over-representation of patients with prostheses relative to the general population of upper limb amputees^39^. This might have led to a selection bias since patients having a myoelectric prosthesis must be able to control their residual limb muscles, which is believed to relate to the ability to perform PLM. However, we did not find any relationship, probably because of the absence of interference between myoelectric prosthesis and PLM control^26^. All our patients clearly expressed that prosthesis control “had nothing to do with PLM control”. Moreover, even patients using another prosthesis or no prosthesis at all need to have some level of control over the residual limb muscles in order to move their residual limb. In the study of Bouffard and colleagues^26^, some patients described that PLM can interfere with myoelectric prostheses, for example when trying to control their prosthesis hand using PLM. This confusion might be involuntarily maintained by health professionals who advise patients to use PLM to control prosthetic movements, in particular after below-elbow amputation. Therefore, health professionals should better differentiate (learned) prosthesis control and (natural) PLM execution.

The third aim of this study was to investigate the potential effects of a daily PLM training in 5 above-elbow amputees without targeted muscle reinnervation. PLM training seems to improve both PLM endurance and speed and, accordingly, the patients reported less difficulty in PLM execution. The absence of improvement for 2 of the patients with more than a year between two recording sessions without training is in line with previous results^31^ showing the absence of a relation between PLM capacity (number of different PLM, peak velocity) and the time elapsed since amputation. The present study suggests that there is no systematic evolution of PLM, neither positive nor negative, when PLM are not practiced. Yet, although 2 months of training increased the PLM performance, they did not normalize with respect to intact-limb movements; they were still slower. To date it is not clear whether a frequent use of PLM (e.g. for daily prosthesis control) can normalize PLM execution.

Interestingly, the increasing residual limb tension (visibly expressed by tremor or expanding contractions), often observed before blocking of the PLM, coincided with the reported increase in execution difficulty. This shows that PLM perception may be (partly) related to somatosensory feedback from the residual muscles. The PLM training delayed the increase in both muscle tension and effort sensation, and thus increased the PLM endurance. In agreement with other studies^33,38^, the training tolerance was good and did not provoke phantom or residual limb pain. This is encouraging for the potential use of PLM for prosthetic control.

Despite the presumed cortical reorganization after amputation^40–42^, the presence of PLM shows that the lost limb is still represented in the primary sensorimotor areas. An internal forward model for the absent limb seems to be preserved, despite the absence of proprioceptive and visual feedback from the concerned limb^43^. The primary motor area can thus send motor commands to the lost limb as it used to do before amputation, but now targeting residual limb muscles (perhaps due to peripheral sprouting^44,45^), leading to motor commands for the phantom limb that are (at least partly) like those for intact limbs.

Motor commands to intact limbs in healthy persons include muscle synergies. These are generally defined as coherent activation, in space and time, of a group of muscles^46^ around one or two articulations. For example, it is shown that the central nervous system controls both intrinsic and extrinsic hand muscles as a muscle synergy^47^. So, for below-elbow amputees, the residual (forearm) muscle activity associated to PLM execution might partly be due to natural muscle synergies. Upper-limb muscles, when active at the same time as hand and finger muscles, usually serve as support to keep the end effector at a required position in space. If residual muscle activity is due only to such synergy, one could expect tonic muscle activity instead of the phasic residual (upper arm) muscle activities found for individual phantom finger movements in above-elbow amputees^19^. However, global supporting contraction schemes (i.e., muscle contractions of proximal residual muscles acting in synergy with movements of the -now missing- distal limb), in phase with the PLM, may still contain information on the executed PLM. Independently of the underlying neural mechanism of the recorded EMG activity associated to PLM, the more controllable the phantom limb, the higher the probability that these EMG recordings are strong and contain characteristic information about the PLM.

Our recent study^31^ identified characteristics of intact limb motor control in the kinematics of PLM. This is significant since, even for above-elbow amputees, it strongly suggests that PLM are naturally controlled without any need for learning. This is not the case for the isolated muscle-activation levels that must be produced for the classical way of controlling myoelectric prostheses. The observation that all patients using a myoelectric prosthesis made a distinction between the control of their prosthesis and the control of PLM, clearly shows that the motor commands differ between the two control modes^26^. Indeed, natural motor control focusses on the movement production rather than on isolated muscle-activation levels. The fact that, even for above-elbow amputees, the residual limb EMG patterns associated with PLM vary as a function of the type of executed phantom finger and hand movements^9,11,13^, suggests that PLM may potentially be used for more intuitive prosthesis control.

In conclusion, the present results are very encouraging for the development of PLM-based prosthesis control. The development of such control approaches depends inevitably on the progress in the -as yet limited- performance and robustness of pattern recognition techniques and on EMG measurement techniques^1^, especially in the complex case of transhumeral amputations. However, our findings support that, from an epidemiological point of view, PLM-based prosthesis control could be feasible, even if this is not yet practically possible with present day technology without the help of surgical interventions. Indeed, the fact that many above- and below-elbow amputees can produce PLM involving phantom hand and fingers is a positive point for polydigital prosthesis control; pain does not prevent the variety of PLM a patient can produce; and PLM do not vanish with time, even when not practiced for a long time. Moreover, training allows patients to increase both PLM endurance and speed, which means that the daily use of PLM for controlling prostheses will probably further increase phantom mobility capacity. While it will now be necessary (i) to characterize the PLM-related EMG activity to ensure its coherence with the perceived sensations, and (ii) to decode these signals in a robust way (especially in above-elbow amputees, with the additional perturbation generated by the wearing of the prosthesis over the residual limb), the present results stimulate the development of a PLM-based control mode of myoelectric prostheses that may become intuitive, natural, and potentially increase the degrees of freedom without necessarily using surgery.

## Methods

### Participants

All adult patients admitted to or followed by the Physical Rehabilitation Center between June 2013 and July 2017 after major acquired upper limb amputation participated in a semi-directed interview. No patient had undergone surgery for targeted muscle reinnervation. The elapsed time since amputation, the aetiology and the laterality as well as the reason for admission or consultation were not considered for inclusion. Poor understanding of the French language was the only criterion of exclusion. None of the patients reported any history of psychiatric disease. The present study is based on 76 patients (12 females), aged from 18 to 82 years (48 ± 12 years, mean ± sd). General patient characteristics are described in Table 2. Thirty-seven patients were above-elbow and 39 below-elbow amputees. Median time post-amputation was 4.7 years (total range: 1 month to 52 years). Nine patients had multiple-limb amputations (5 bilateral, 3 quadri-amputation and 1 both an upper and lower limb). Thirty-four patients had a medical specific treatment for phantom pain and some also for residual-limb pain.

**Table 2:** Descriptive statistics of patient characteristics.

Gender	12♀ 64♂
Age at the time of interview (mean ± sd)	48± 12 years
Age at the time of amputation (mean ± sd)	36± 12 years
Time post-amputation (median; 1^st^ – 3^rd^ quartile)	4.7; 0.4–15 years
Amputation on the dominant side	44
Bilateral amputation	8
**Type of amputation**	
*Above elbow*	
-Scapulo-thoracic disarticulation	2
-Upper arm	34
-Elbow disarticulation	1
*Below elbow*	
-Forearm	28
-Wrist disarticulation	11
**Aetiology**	
-Trauma	55
-Electrocution	4
-Neurological pathology	3
-Local infection	4
-General infection	3
-Vascular pathology	2
-Tumour	2
-Burn	3
**Use of prosthesis**	
-No	28
-Fitting in progress	22
-Myoelectric prosthesis	36
-Mechanical prosthesis	12
-Aesthetic prosthesis	9

Either number of occurrence or mean and standard deviation (sd) are provided, except for time post-amputation since these data were not normally distributed; median value and interquartile ranges are thus provided for this variable. Note that many patients use several types of prostheses.

Five of these patients voluntarily participated in a phantom-mobility training protocol. The inclusion required a unilateral above-elbow amputation dating back more than 6 months, capacity to generate PLM and declared motivation to train daily at home by repeating all the phantom movements the patient could execute.

Most of the questioning was part of the normal intake interview with patients. The questions, specifically based on non-painful sensations and the mobility of the phantom limb, were approved by the Local Ethical Committee of the Physical Rehabilitation Center. The training protocol was approved by an ethics committee (“Conseil d’Evaluation Ethique pour les Recherches en Santé” de Paris Descartes, N° 2016 − 57) and the study was performed in accordance with the Declaration of Helsinki. All the subjects gave written informed consent prior to participating.

### Protocol

A semi-directed interview based on a questionnaire was carried out. The first part of the interview concerned demographic data: Time elapsed since amputation, the aetiology, medical treatment and reason for either admission or consultation. The second part of the questionnaire contained themes related to the manifestations of the phantom limb (if present) and of the residual limb. Besides phantom mobility, we explored other phantom limb manifestations through questioning about both painless and painful sensations. Information about size, posture and form of phantom limbs were obtained. Patients with voluntary phantom limb mobility were encouraged to describe the types of movements they could produce as well as whether phantom or residual limb pain and/or fatigue potentially limited their production. For the mobility, we had predetermined 13 different types of movements (9 concerning the hand; 3 for the wrist; 1 for the elbow) and conducted a standardized interview asking the patients whether or not they could perform each of these movements. To confirm the described movement type, amplitude and speed, patients were encouraged to demonstrate each movement by mimicking it simultaneously with the intact limb. Since the 8 patients with bilateral arm amputations could not imitate the PLM, we based the results on their oral description of phantom mobility. Finally, for patients using a prosthesis, information was obtained about the type of prosthesis and its wear-time. Also, they were asked whether they thought that they controlled their prosthesis with muscle activity associated to PLM-execution or not. For those who had never worn, or no longer wore, a prosthesis, they were asked to specify the reason. As the present paper focusses on phantom limb mobility, other phantom or residual limb sensations (painful or painless) will only be shortly mentioned but not elaborated unless related to phantom mobility.

Five patients with a unilateral above-elbow amputation and phantom mobility, volunteered for a training protocol which consisted of training at home on a daily basis during 1 or 2 months all their possible phantom movements. They were asked to make cyclic PLM with as many cycles as possible without blocking (up to 10 or 15 if possible). If the patients felt that they were able to perform a new PLM (i.e., one they were previously unable to execute), they should also train that movement. They were instructed to stop immediately if the training induced pain. During the training period a weekly phone interview was organized in order to evaluate tolerance and observance. Two particularly motivated patients were asked to rate on a daily basis the difficulty of PLM execution on a Borg Rating of Perceived Exertion (RPE) Scale (from 6 to 20)^34^. Yet, for one of these two patients, the Borg rating was found to be difficult to apply. Therefore, the difficulty of PLM execution was appreciated through the number of cycles he could execute before eventual blocking of the PLM occurred as well as the first cycle that he experienced as difficult to execute.

Their PLM capacities were video-recorded directly before and after the training period. During these recordings, as during the training period, the patients were asked to make cyclic phantom movements, by performing as many cycles as possible without blocking (they were stopped at 15). In order to visualize the movements on the video, patient synchronously mimicked the PLM with the intact limb. In this protocol we evaluated the number of different types of PLM, the endurance of PLM execution (through the number of cycles) and the speed of PLM execution (through cycle duration). To check whether the modifications were due to PLM training rather than to the elapsed time between the two sessions, we compared for two patients (P3 and P5) who had participated in an earlier phase of the study, their performance during the very first time we recorded their PLM with those during the pre-training session. Twelve months separated these recordings for patient P3 and 30 months for P5. Finally, in order to evaluate whether the PLM after training normalized with respect to intact limb movements, we asked the same two participants (P3 and P5) to perform with their intact limb one of the PLM types of movements at maximal speed; P3 made a series of 10 cyclic pinch opening/closing, and P5 wrist flexion/extension. We qualitatively compared the mean cycle duration of the intact limb movements with that of the corresponding PLM.

### Analysis

Concerning the questionnaire, for the 8 patients with bilateral upper limb amputations, if the number and types of the PLM were not symmetric, we took into account the most mobile phantom limb for the study. For each patient and for each phantom segment or joint (i.e., fingers, wrist and elbow) we determined the number of different achievable voluntary PLM. The total number of different voluntary PLM over all upper limb segments and joints will be called “mobilization capacity”. Linear regression was determined in order to explore whether and how the mobilization capacity evolved over the elapsed time since amputation. For this analysis, phantom elbow movements were not taken into account since below-elbow amputees still have this articulation. The mobilization capacity being non-normally distributed (Test of Lilliefors, p < 0.05), in order to test whether the amputation level influences the mobilization capacity, we applied a Mann-Whitney test to compare the mobilization capacity between above- and below-elbow amputees (again excluding the elbow articulation), and confirmed the results with Fisher’s exact test for which the power is given. Finally, a Mann-Whitney test was applied to compare the mobilization capacity between users of myoelectric prostheses and those using no prosthesis or another type of prosthesis.

Concerning the training protocol, the videos allowed to identify the type of PLM (and thus the number of different types), to count the number of cycles for each type of phantom movement and to determine the duration of one series of cyclic movements. Dividing the total duration by the number of cycles gave the mean cycle duration for each type of phantom movement. For each participant, we averaged the number of cycles as well as the cycle durations over all types of their phantom movements. Because of the small sample, the PLM performance before the training period was statistically compared to those after the training period with help of Wilcoxon tests for paired samples. Then, we compared for each of two patients (P3 and P5) who had participated in an earlier phase of the study, their performance during the first time we recorded their PLM with those during the last recording session before the training period with a Wilcoxon test for paired samples, taking their individual types of PLM as cases and the two sessions as repeated measures. For all statistical tests, corrected for multiple comparisons, the significance threshold was chosen at p = 0.05.
